# A Review on the Clinical Diagnosis of Multiple System Atrophy

**DOI:** 10.1007/s12311-022-01453-w

**Published:** 2022-08-19

**Authors:** Iva Stankovic, Alessandra Fanciulli, Victoria Sidoroff, Gregor K. Wenning

**Affiliations:** 1 Neurology Clinic, University Clinical Center of Serbia, Faculty of Medicine, University of Belgrade, Belgrade, Serbia; 2 Department of Neurology, Medical University of Innsbruck, Innsbruck, Austria

**Keywords:** Multiple system atrophy, Neurogenic orthostatic hypotension, Urogenital failure, Parkinsonism, Cerebellar ataxia

## Abstract

Multiple system atrophy (MSA) is a rare, adult-onset, progressive neurodegenerative disorder with major diagnostic challenges. Aiming for a better diagnostic accuracy particularly at early disease stages, novel Movement Disorder Society criteria for the diagnosis of MSA (MDS MSA criteria) have been recently developed. They introduce a neuropathologically established MSA category and three levels of clinical diagnostic certainty including clinically established MSA, clinically probable MSA, and the research category of possible prodromal MSA. The diagnosis of clinically established and clinically probable MSA is based on the presence of cardiovascular or urological autonomic failure, parkinsonism (poorly L-Dopa-responsive for the diagnosis of clinically established MSA), and cerebellar syndrome. These core clinical features need to be associated with supportive motor and non-motor features (MSA red flags) and absence of any exclusion criteria. Characteristic brain MRI markers are required for a diagnosis of clinically established MSA. A research category of possible prodromal MSA is devised to capture patients manifesting with autonomic failure or REM sleep behavior disorder and only mild motor signs at the earliest disease stage. There is a number of promising laboratory markers for MSA that may help increase the overall clinical diagnostic accuracy. In this review, we will discuss the core and supportive clinical features for a diagnosis of MSA in light of the new MDS MSA criteria, which laboratory tools may assist in the clinical diagnosis and which major differential diagnostic challenges should be borne in mind.

## Introduction

Multiple system atrophy (MSA) is a rare, fatal, rapidly progressive neurodegenerative disease of adulthood, characterized by severe autonomic failure, poorly L-Dopa-responsive parkinsonism, cerebellar ataxia, and pyramidal signs in any combination [1]. It is classified as MSA-parkinsonian (MSA-P), if parkinsonism prevails, and MSA-cerebellar (MSA-C), if cerebellar features predominate. The MSA-C variant typically outnumbers the MSA-P one in East Asian natives: large cohorts of Japanese, Korean, and Chinese reported a MSA-C phenotype in up to 73% of examined patients [2–6], with some exceptions in the case of late-onset MSA and one Chinese and Korean cohort [4, 7, 8]. By contrast, in European, American, and Moroccan cohorts, MSA-P was the most commonly censored phenotype [9–11] [12].

With its variable clinical presentation, MSA represents a major diagnostic challenge throughout its disease course. Due to isolated autonomic complaints at prodromal stages, patients with MSA may refer to other specialists such as cardiologists or urologists first. When motor symptoms develop, but are still very mild, MSA may not be distinguishable from Parkinson’s disease (PD) or other causes of sporadic adult-onset cerebellar ataxia (SAOA). Overlapping features with other atypical parkinsonian disorders, such as dementia with Lewy bodies (DLB) or progressive supranuclear palsy (PSP), eventually impact on MSA diagnostic accuracy also at more advanced disease stages [13].

Following Quinn’s effort [14] to rationalize the clinical diagnosis of MSA, consensus conferences were held in 1998 [15] and 2008 [16] to integrate the diagnostic criteria with the latest advances in MSA clinical, pathological, and biomarker research. Recent clinicopathological series highlighted however an overall suboptimal diagnostic accuracy, low sensitivity at early disease stages [13, 17, 18], and other critical issues of the latest MSA criteria set [19], which eventually prompted the recent revision of MSA diagnostic criteria endorsed by the International Parkinson and Movement Disorders Society (MDS) [20].

In the present review, we first provide an overview of the MSA core clinical features, as well as supportive motor and non-motor features, which should raise a suspicion of MSA on clinical grounds. Afterwards, we discuss the new set of MDS MSA diagnostic criteria, with an in-depth focus on ancillary laboratory findings and strategies to distinguish MSA from its most frequent look-alikes.

## Core Clinical Features of MSA

### Autonomic Failure

Up to one-third of patients presenting with isolated autonomic failure [21] may phenoconvert over years into another central nervous system disorder including MSA [22–24]. Between 5 and 30% of MSA patients may in fact present with urogenital and cardiovascular autonomic symptoms several years before any other motor symptom develops [10, 25, 26].

Bladder function consists of a *storage phase*, in which the urethral sphincter is active and the bladder detrusor inhibited, and a *voiding phase*, during which the contemporary activation of the bladder detrusor and relaxation of the urethral sphincter favors bladder emptying. Disorders of neural control of urinary function may result in disturbances of the storage phase, such as urinary urge incontinence, and voiding difficulties with interrupted stream and increased postvoid urinary residual volume. While disturbances of the storage phase have a major impact on self-confidence and overall quality of life, voiding difficulties put affected patients at increased risk of urinary tract infections, hydronephrosis, and secondary kidney failure [27].

In 73 to 87% of all MSA patients, disturbances of the storage phase develop early in the disease course due to detrusor overactivity and external sphincter weakness [28, 29]. As the disease progresses, sphincter-detrusor dyssynergia arises, with increasing voiding difficulties, postvoid urinary residual volume, typically > 100 mL, and recurrent urinary tract infections [30]. Erectile dysfunction occurs very early in the disease course and accompanies bladder dysfunction in virtually all male MSA patients [9, 10]. Questionnaire-based studies also found reduced genital sensitivity in 47 to 56% of female MSA patients [31, 32]. In both sexes, sexual dysfunction is due to degenerative changes occurring in the spinal sacral region and may precede MSA motor onset by months or years [10, 33, 34].

Cardiovascular autonomic failure in MSA develops after neurodegenerative changes of the central autonomic nervous system (ANS), including brainstem autonomic nuclei and pre-ganglionic sympathetic fibers to the heart and blood vessels, and typically results in neurogenic orthostatic hypotension (OH). Neurogenic OH is defined by a sustained systolic blood pressure fall ≥ 20 mmHg within 3 min of head-up tilt or active standing, usually accompanied by a diastolic blood pressure fall ≥ 10 mmHg, which, however, remains unspecific if isolated [35]. Due to efferent baroreflex dysfunction [36], patients with neurogenic OH typically show a pathological blood pressure counter-regulation during cardiovascular autonomic function tests such as the Valsalva maneuver and, despite severe falls in blood pressure upon standing, very blunted or sometimes missing compensatory heart rate increases. An increase in heart rate < 0.5 beats per minute/mmHg of systolic blood pressure fall after 3 min of head-up tilt or active standing reliably distinguishes neurogenic OH from non-neurogenic causes of OH (sensitivity: 91.3%; specificity: 88.4% for head-up tilt test; sensitivity: 79% for standing test), such as volume depletion or blood pressure–lowering drugs [37, 38]. At established disease stages, neurogenic OH occurs in 75 to 81% of MSA patients [9, 39, 40], with possibly higher frequencies and severity degrees in the MSA-C than the MSA-P variant [41﻿].

The 2008 MSA diagnostic criteria had introduced higher cut-off values to diagnose neurogenic OH in the setting of MSA (≥ 30 mmHg systolic or ≥ 15 mmHg diastolic within 3 min of an orthostatic challenge), but these did not significantly improve the specificity for a MSA diagnosis, while worsening its sensitivity [42]. By contrast, at earlier disease stages, neurogenic OH may develop beyond the classical 3 min of orthostatic challenge, a phenomenon called delayed OH. Prolonging the passive or active orthostatic challenge from 3 to 10 min increases the sensitivity for neurogenic OH by 18% and it is therefore recommended at the time of the first diagnostic work-up [43].

Every second person with MSA and neurogenic OH shows accompanying phenomena, such as postprandial hypotension [44] and supine and nocturnal hypertension [45, 46]. While such additional features have major implications on the disease prognosis [47], their differential diagnostic yield remains low due to their high prevalence also in patients with autonomic failure due to other parkinsonian disorders [48].

### Parkinsonism

Parkinsonism in MSA is usually symmetric, rapidly progressive, and moderately to poorly responsive to L-Dopa. Due to its axial involvement, patients experience early postural instability associated with falls. Rapid progression leads to a shorter latency to disease milestones in MSA; as early as after 3 years from onset, one-third of patients with MSA require walking aids, and after 5 years, up to 60% of patients become wheelchair-bound [1]. Early postural instability is specific but moderately sensitive for MSA diagnosis [18, 49]. Overall, MSA-C patients have a higher Hoehn and Yahr stage than MSA-P ones due to unsteadiness caused by cerebellar features [50].

Patients with MSA have in general poorer response to L-Dopa compared to patients with PD. Poor L-Dopa responsiveness is defined either by history taking or as less than 30% improvement on the MDS-UPDRS III on up to 1000 mg L-dopa if needed or tolerated for at least a month. A retrospective study on a mixed cohort of 100 clinically and postmortem diagnosed MSA patients reported that, based on the subjective assessment of the patient or caregiver, 8% had an excellent response to L-Dopa (> 70% benefit), 12% had a good response (50 to 69% benefit), 6% had a moderate response (30 to 49% benefit), and 74% reported a poor response to L-Dopa (< 29% benefit) [50]. A proportion of MSA patients (42 to 57% of MSA-P and 13 to 25% of MSA-C patients) may initially show a beneficial, but transient, response to L-Dopa, which diminishes after an average of 3.5 years [51, 52]. Given that the majority of MSA patients experience side effects upon L-Dopa administration, and that an initial poor response may refrain a clinician from introducing a regular L-Dopa therapy, an acute L-Dopa challenge test should be avoided in clinical practice [53].

Motor complications include wearing-off fluctuations in 23%, off-period dystonia in 20%, on–off fluctuations in 14%, and peak-dose dyskinesia in 11% of moderately advanced MSA patients [51]. Dyskinesia in patients with MSA is usually focal, asymmetric, and dystonic affecting the craniocervical or hands/feet regions; this contrasts with the generalized choreatic limb dyskinesia typical of patients with PD [50, 54]. Patients with young-onset MSA more frequently have a good response to L-Dopa, L-Dopa-induced dyskinesia, and dystonic features compared to patients with the classic disease onset in the 6^th^ decade of life [55]. Focal dystonic postures affecting the face, neck, or limbs prior to the initiation of L-Dopa were recorded in up to half of MSA patients, even preceding parkinsonism in some [33].

### Cerebellar Syndrome

Cerebellar features, most commonly a broad-based ataxic gait, occur in 36 to 64% of all MSA patients [9, 25, 26, 33, 39]. Other cerebellar signs encompass limb ataxia (47 to 53% of cases), scanning dysarthria (49 to 69%), and postural and intention tremor (24 to 56%) as well as cerebellar oculomotor abnormalities, such as sustained gaze-evoked horizontal, positional downbeat nystagmus, and saccadic hypermetria (23%) [9, 29, 33, 56]. If considering MSA-C patients only, gait ataxia is present in virtually all patients, limb ataxia in 87 to 94%, postural tremor in 45%, and oculomotor abnormalities in 38 to 41% [9, 10, 29]. Some clinical features, such as the *phalanx sign*, which is tested with 5 nose-to-examiner’s finger repetitions and indicates limb dysmetria if the proband’s finger touches the distal interphalangeal joint or beneath, are bedside semiological tools helpful to distinguish MSA-C from SAOA, when no clear parkinsonism or autonomic failure is present [57].

### Supportive Motor and Non-motor Features for MSA Diagnosis

Supportive clinical features, previously termed “red flags,” include motor and non-motor features suggestive of MSA that usually become manifest after several years into the disease course (Table 1). Neuropathological studies showed that the frequency of such features did not differ in the first 3 years from disease onset between MSA and other parkinsonian disorders such as Lewy body disease and PSP, overall indicating their low diagnostic sensitivity in early disease stages [13]. However, if a patient develops at least one out of orofacial dystonia, inspiratory sighs, contractures of hands or feet, polyminimyoclonus, severe dysarthria, pathologic laughter or crying, and cold hands and feet, this person is 8.8 times more likely to have MSA-P and 7 times more likely to have MSA-C than a Lewy body disorder (sensitivity: 74%, specificity: 88.5%) [13]. If any out of orofacial dystonia, inspiratory sighs, contractures of hands and feet, jerky myoclonic postural/action tremor, poliminimyoclonus, severe dysphonia, and snoring are present, the chance that this person has MSA-P or MSA-C is respectively 4.3 or 3.1 times higher than PSP (sensitivity for MSA-P: 72.8%, for MSA-C: 66.7%, specificity: 76.9%) [13].

**Table 1:** MDS criteria for the diagnosis of MSA [20]

Neuropathologically established MSA	• Widespread and abundant CNS α-synuclein positive glial cytoplasmic inclusions associated with neurodegenerative changes in striatonigral or olivopontocerebellar structures [58]
Essential features for clinical MSA diagnosis	• Sporadic, progressive, adult (> 30 years) onset disease
Clinically established MSA	• Autonomic dysfunction defined as at least one of voiding difficulties with postvoid urinary residual volume > 100 ml, urinary urge incontinence or neurogenic OH^&^ associated with at least one of poorly L-Dopa-responsive parkinsonism and cerebellar syndrome (≥ 2 features^*^) • ≥ 2 supportive clinical features • ≥ 1 brain MRI marker • Absence of exclusion criteria
Clinically probable MSA	• At least two of autonomic dysfunction defined as at least one of voiding difficulties with postvoid urinary residual volume, urinary urge incontinence or delayed neurogenic OH^#^, parkinsonism, and cerebellar syndrome (≥ 1 feature^*^) • ≥ 1 supportive clinical feature (excluding erectile dysfunction alone) • Absence of exclusion criteria
Possible prodromal MSA	• At least one of polysomnography-proven RBD, delayed neurogenic OH^#^, and urogenital failure defined as at least one of erectile dysfunction before age of 60 years with voiding difficulties and with postvoid urinary residual volume > 100 ml or urinary urge incontinence • Either subtle cerebellar or subtle parkinsonian signs or both • Absence of exclusion criteria
Supportive clinical features	• Motor features occurring within 3 years from onset such as rapid progression, postural instability, severe speech impairment, and severe dysphagia as well as features appearing at any time during the disease course including craniocervical dystonia induced or exacerbated by L-Dopa in the absence of limb dyskinesia, unexplained Babinski sign, jerky myoclonic postural or kinetic tremor, postural deformities • Non-motor features including stridor, inspiratory sighs, cold discolored hands and feet, erectile dysfunction and emotional incontinence
Brain MRI markers	• For MSA-P: atrophy of putamen, middle cerebellar peduncle, pons and cerebellum, hot cross bun sign, and increased diffusivity of putamen and middle cerebellar peduncle • For MSA-C: atrophy of putamen, middle cerebellar peduncle and pons, hot cross bun sign, and increased diffusivity of putamen
Exclusion criteria	• For clinically established and clinically probable MSA: sustained beneficial response to L-Dopa and unexplained anosmia on olfactory testing • For possible prodromal MSA: at least one of unexplained anosmia on olfactory testing or abnormal cardiac sympathetic imaging (^123^I-MIBG-scintigraphy) • For all clinical categories: typical clinical and brain MRI features of MSA-mimicking conditions such as PD, DLB, PSP, genetic, and other causes of ataxia

^*^Cerebellar features: gait ataxia, limb ataxia, cerebellar dysarthria, and oculomotor features; ^#^delayed neurogenic OH: ≥ 20/10 mmHg blood pressure drop and ΔHR/ΔSBP ratio < 0.5 bpm/mmHg within 10 min in an upright position; ^&^neurogenic OH: ≥ 20/10 mmHg blood pressure drop and ΔHR/ΔSBP ratio < 0.5 bpm/mmHg within 3 min in an upright position

*bpm* beats per minute, *CNS* central nervous system, *DLB* dementia with Lewy bodies, *HR* heart rate, *MRI* magnetic resonance imaging, *MSA-C* MSA-cerebellar type, *MSA-P* MSA-parkinsonian type, *MSA* multiple system atrophy, *OH* orthostatic hypotension, *PD* Parkinson’s disease, *PSP* progressive supranuclear palsy, *RBD* REM sleep behavior disorder, *SBP* systolic blood pressure

The European MSA study group applied a checklist of supportive clinical features for a diagnosis of MSA to patients with probable MSA-P or PD. Only features with a specificity > 95% were further categorized into six domains: early instability, rapid progression, abnormal postures, bulbar dysfunction, respiratory dysfunction, and emotional incontinence. The presence of supportive clinical features from ≥ 2 categories showed good diagnostic accuracy (sensitivity: 84%, specificity: 98%). If this criterion were applied to 17 patients with possible MSA-P, 76% of them would have been correctly diagnosed as probable MSA-P on average 16 months earlier [59].

### MDS Criteria for the Diagnosis of MSA

The new MDS MSA criteria [20] have been developed to increase the diagnostic accuracy of MSA, particularly in the early disease stages (Table 1). These criteria include four levels of diagnostic accuracy: neuropathologically established MSA, clinically established MSA, clinically probable MSA, and possible prodromal MSA. Neuropathologically established MSA is a postmortem-confirmed diagnostic category, which equals the definite MSA category of the 2008 consensus criteria [16]. The clinically established MSA category is designed to secure maximum specificity, with acceptable sensitivity, while the clinically probable MSA category allows for a balanced sensitivity and specificity. Possible prodromal MSA is a pure research category designed to capture patients in the prodromal phase of the disease. Clinical MSA categories are composed of essential and core clinical features, supportive motor and non-motor features, and absence of exclusion criteria. The presence of the MSA-specific brain MRI marker is required for the clinically established category only, while the clinically probable category is based exclusively on clinical features. The diagnostic accuracy of the MDS MSA criteria will be verified in a prospective clinical multicenter study.

Essential features for a diagnosis of MSA include an onset after the age of 30 years, a progressive course, and a negative family history. The former division into the MSA-P and MSA-C categories has been retained. Core clinical features for clinically established MSA and clinically probable MSA include cardiovascular or urogenital autonomic failure, parkinsonism, and cerebellar ataxia stratified according to their diagnostic yield into one of the two categories. Specific but moderately sensitive clinical features are a benchmark of clinically established MSA, whereas less specific but more sensitive features occurring usually earlier in the disease course define the clinically probable MSA category. Unexplained voiding difficulties with postvoid residual urinary volume > 100 ml are necessary for the diagnosis of clinically established MSA, while the finding of postvoid residual urinary volume < 100 ml is sufficient for the diagnosis of clinically probable MSA. L-Dopa-unresponsive parkinsonism is required for the diagnosis of clinically established MSA, but a patient with a good or moderate response to L-Dopa may be still diagnosed with clinically probable MSA. At least two cerebellar features are required for a diagnosis of clinically established MSA, yet a single feature is required for clinically probable MSA. At least two supportive clinical (either motor or non-motor) features are necessary for diagnosing clinically established MSA, and only one for clinically probable MSA (excluding isolated erectile dysfunction). Autonomic failure is mandatory for the diagnosis of clinically established MSA, whereas any combination of two out of three core clinical features including that of the cerebellar syndrome and parkinsonism without autonomic failure allows for the diagnosis of clinically probable MSA. Exclusion criteria for clinically established and clinically probable MSA include the presence of sustained beneficial response to L-Dopa, unexplained anosmia on olfactory testing, and typical clinical and brain MRI features of MSA-mimicking conditions such as PD, DLB, PSP, genetic, or other causes of ataxia.

The possible prodromal MSA category is devised to diagnose patients at the earliest stages of the disease, when non-motor symptoms are predominant and associated with only mild motor symptoms. This category in particular needs validation in future studies. The entry criteria for this category are polysomnography-proven REM sleep behavior disorder (RBD), delayed neurogenic OH within 10 min in the orthostatic position, and urogenital dysfunction. In an attempt to distinguish patients with possible prodromal MSA from patients with prodromal PD, an absence of either unexplained anosmia on olfactory testing or abnormal cardiac sympathetic imaging (^123^I-MIBG-scintigraphy) or both is required. Other exclusion criteria for this category are essentially the same as for other clinical categories.

### Supportive Laboratory Findings for MSA Diagnosis

Although the diagnosis of MSA remains primarily a clinical exercise, brain MRI and evaluation of postvoid residual urinary volume using bladder sonography, in-and-out catheterization, or urodynamics are now required for the clinical diagnosis of MSA, whereas polysomnography is required for the diagnosis of possible prodromal MSA, if RBD is the presenting symptom. Other ancillary investigations as shown in Table 2 may be helpful to increase the diagnostic accuracy at early disease stages [20] and often contemporarily identify individual therapeutic needs. However, one should keep in mind that these investigations are formally not part of the MDS MSA criteria [20] due to their limited diagnostic yield, limited availability, and a lack of validation. Future studies will clarify which additional laboratory tests yield sufficient accuracy for the MSA diagnosis, which will justify their inclusion in future revisions of the MSA diagnostic criteria [20].

**Table 2:** Supportive laboratory markers of MSA diagnosis (modified from [20])

• Brain MRI markers (for possible prodromal MSA only) including atrophy of putamen, MCP, pons and cerebellum, hot cross bun sign, and increased diffusivity of putamen and MCP
• Normal cardiac sympathetic imaging and (for clinically established and clinically probable MSA only)
• Polysomnography-proven RBD (for clinically established and clinically probable MSA only)
• FDG-PET hypometabolism of putamen, brainstem, and cerebellum for MSA-P and FDG-PET hypometabolism of putamen for MSA-C
• Supine plasma norepinephrine level > 100 pg/ml associated with neurogenic OH measured using high-performance liquid chromatography with electrochemical detection after 10 min in supine position
• Detrusor hyperactivity with impaired contraction or detrusor sphincter dyssynergia on urodynamic testing
• Abnormal sphincter EMG with manual analysis including late components defined as > 20% of MUPs with duration > 10 ms or the average duration of MUPs > 10 ms
• α-synuclein oligomers detected in CSF by PMCA or RT-QUIC
• Increased NfL in CSF or plasma detected by ELISA

*CSF* cerebrospinal fluid, *FDG-PET* fluorodeoxyglucose-positron emission tomography, *MCP* middle cerebellar peduncle, *MRI* magnetic resonance imaging, *MSA-C* MSA-cerebellar type, *MSA-P* MSA-parkinsonian type, *MSA* multiple system atrophy, *MSA* multiple system atrophy, *MUP* motor unit potential, *NfL* neurofilament light chain, *OH* orthostatic hypotension, *PMCA* protein misfolding cyclic amplification, *RT-QUIC* real-time quaking-induced conversion

At least one MSA-typical finding at 1.5 T or 3 T brain MRI is required for a clinically established diagnosis of MSA (Table 1). The diagnostic yield of the different MRI findings varies depending on the prevalent motor phenotype. While structural brain MRI findings show high specificity for a MSA diagnosis, their sensitivity remains limited, especially at the early disease stage [20]. On the other hand, increased putaminal diffusivity shows an overall high diagnostic accuracy for distinguishing MSA from PD, and increased diffusivity of the middle cerebellar peduncle for differentiating MSA from PSP [60]. Diagnostic algorithms based on multimodal MRI approaches that included observer-independent brain volumetry, diffusion-weighted imaging, and iron-sensitive sequences recently showed an excellent accuracy for a MSA diagnosis [60]. Harmonization of protocols for MRI execution and analysis will likely promote their implementation outside of specialized centers in the future.

Functional neuroimaging studies may be also implemented in the diagnostic work-up of MSA, even though are not mandatory for its diagnosis. Phenotype-dependent varying degrees of the putamen, pons, and cerebellar hypometabolism on brain FDG-PET are highly suggestive of MSA in patients presenting with parkinsonism or sporadic late-onset cerebellar ataxia [60], even though cerebellar hypometabolism may also occur in other causes of genetic or acquired cerebellar ataxia.

Cardiac ^123^I-metaiodobenzilguanidine (MIBG) SPECT is an instrumental marker of cardiac sympathetic innervation, and decreased cardiac MIBG uptake indicates degeneration of postganglionic sympathetic fibers to the heart. Studies from different research groups indicated that cardiac MIBG-SPECT may support the distinction of MSA from PD, because MSA is characterized by a pre-ganglionic pattern of ANS lesion and normal cardiac MIBG uptake. Nevertheless, heterogeneous patterns of cardiac tracer uptake may occur in both diseases [61] possibly due to different spreading patterns of the neurodegenerative process in single patients [62], limiting the diagnostic yield of cardiac MIBG-SPECT in differentiating MSA from PD. Drugs interfering with noradrenaline transport and vesicular storage, concomitant heart disease, and other frequent causes of small fiber neuropathy may further impact on cardiac MIBG diagnostic accuracy in clinical practice.

Supine plasma noradrenaline levels derive from noradrenaline spill-over from sympathetic nerve endings and represent another “wet” biomarker of postganglionic sympathetic nerve integrity. In patients with MSA, supine plasma noradrenaline levels are indeed usually normal (> 100 pg/ml), while in patients with isolated neurogenic OH, who later phenoconverted to PD or DLB, these were reported to be low, again consistent with a pre-ganglionic pattern of ANS lesion in MSA versus a postganglionic one in PD and DLB [22, 24].

Recent biomarker studies also showed striking differences between the cerebrospinal fluid (CSF) profiles of patients with MSA, which is characterized by oligodendroglial accumulation of α-synuclein, versus those of patients with PD or DLB, both characterized by neuronal accumulation of α-synuclein in the so-called Lewy bodies. Increased CSF levels of neurofilament light chain > 1.400 pg/ml accurately distinguish MSA from Lewy body disorders (either PD or DLB) already at prodromal disease stages, in which patients may present with otherwise undistinguishable isolated autonomic failure [53, 63, 64]. At protein misfolding cyclic amplification (PMCA) CSF assays, the differences in α-synuclein strains conformation induce divergent reaction kinetics, with a faster aggregation rate, but overall lower maximum Thioflavin T fluorescence in patients with MSA compared to those with Lewy body disorders, ultimately enabling an accurate distinction between oligodendroglial and neuronal α-synucleinopathies both at established and prodromal disease stages [63, 64].

At the fiberoptic evaluation of swallowing (FEES), more than 90% of patients with MSA display various degrees of laryngeal motion abnormalities, which are otherwise only seldom observed in the setting of PD. FEES findings, whose diagnostic accuracy for MSA diagnosis is being further investigated in a multicenter initiative of the MDS MSA Study group, also provide important information for the therapeutic management of dysphagia and stridor, which often complicate advanced MSA stages.

In order to facilitate the standardization of MSA diagnosis also outside of referral centers, the criteria to diagnose urogenital autonomic failure purposely rely on clinical signs and widely available investigations such as ultrasonography or in-and-out catheterization to determine the postvoid residual urinary volume. Urodynamic, and in some cases video-urodynamic testing, is however the gold standard to separate neurogenic from non-neurogenic causes of lower urinary tract dysfunction, and to identify MSA peculiar patterns, such as insufficient bladder neck tone or detrusor-sphincter dyssynergia, and tailor the therapeutic approach [27]. External anal sphincter denervation at concentric needle electromyography reflects neurodegenerative changes in the sacral Onuf’s nucleus and also points towards a MSA rather than PD diagnosis, if documented early in the disease course [65]﻿. Similar findings may be however observed in patients with PSP or advanced PD as well [66].

### Differential Diagnosis of MSA

Overlapping features of MSA and disorders mimicking MSA that present with autonomic failure, parkinsonism, and cerebellar syndrome result in a common misdiagnosis, particularly in early disease stages. Based on the predominant phenotype, differential diagnoses must be considered in the clinical diagnostic work-up.

### Disorders Presenting with Autonomic Failure

Early onset of autonomic failure distinguishes MSA from PD, in which autonomic failure typically develops at more advanced disease stages [67], and other causes of acquired or inherited cerebellar ataxia that usually display milder forms of autonomic failure, if any [68, 69]. At established disease, the presence of severe and widespread autonomic failure is a core clinical feature of MSA and ultimately differentiates it from other atypical parkinsonian disorders, such as PSP and corticobasal degeneration, for which severe multi-domain autonomic failure represents an exclusion criterion [70–73]. The presence of urinary retention is highly specific of MSA versus PD [41], but less accurate in distinguishing MSA from PSP, in which neurogenic bladder disturbances may also develop [74].

A number of disorders presenting with autonomic failure need to be ruled out for the diagnosis of MSA. The most common cause of peripheral autonomic neuropathy worldwide is diabetes, followed by kidney and liver disorders, amyloidosis, infectious (HIV), and paraneoplastic and autoimmune disorders (such as Sjogren’s syndrome, systemic lupus erythematosus, rheumatoid arthritis), which all need to be considered in the differential diagnosis of patients presenting with isolated autonomic failure. If the origin of OH is determined to be non-neurogenic, polypharmacy, dehydration, and anemia need to be sought for. In patients presenting with a transient loss of consciousness, cardiac and reflex-mediated syncope, epileptic seizures, and non-epileptic spells should be ruled out [19].

Previous pelvic surgery, concomitant prostatic hypertrophy in men, or pelvic floor prolapse in women may exacerbate or mask bladder disturbances of neurogenic origin. In a series of patients with probable MSA, 43% of males had undergone futile prostatic or bladder neck surgery before the correct diagnosis was made [75]. Similarly, other case series showed that surgery for urinary stress incontinence gave unsatisfying results in female MSA patients [75, 76]. The diagnostic yield of isolated sexual dysfunction in identifying patients suffering from incipient MSA remains low due to the high prevalence of non-neurogenic causes of sexual dysfunction in the aging population (e.g., endocrine, vascular). Gastrointestinal disorders as an alternative cause of dysphagia and constipation need to be excluded as well. Hypothyroidism may manifest with hypo/anhidrosis and disordered thermoregulation; cold hands and feet may also occur in the case of peripheral vascular disorders.

### Disorders Presenting with Parkinsonism

Already at symptom onset, patients with MSA show more severe parkinsonism compared to patients with PD (Hoehn and Yahr stage > 2 in 54% of MSA and 21% of PD cases) [77–79]. Although parkinsonian features are often symmetric in MSA, around 40% of patients manifest with persisting unilaterality throughout the disease course [17, 29]. Tremor as a presenting symptom is significantly less frequent in MSA (12.5 to 22%) than in PD (60%) [78, 79], and if “atypical” (irregular postural or kinetic and associated with myoclonic jerk, instead of classic “pill-rolling” tremor) can be useful for differentiating between the two disorders [33]. Rapid progression, defined as reaching Hoehn and Yahr stage ≥ 3 within three years from onset (sensitivity: 69%; specificity: 90%), and early recurrent falls are typical of MSA compared to PD [78, 80]. In contrast to the monotonous and hypophonic speech (hypokinetic dysarthria) of patients with PD, patients with MSA often manifest a slurring cerebellar dysarthria or a quivering, strained, croaky, or high-pitched quality voice defined as a mixed dysarthria of hypokinetic-ataxic-spastic type [50, 81]. If by assigning one point each for the presence of postural instability, neurogenic OH, symptoms of overactive bladder, or urinary retention in the first 2 years from symptom onset, a cumulative score ≥ 2 is reached, MSA may be distinguished from PD with 78% sensitivity and 86% specificity [67]﻿.

Clinical features that argue against MSA diagnosis are sustained L-Dopa response or motor fluctuations with marked peak-dose appendicular dyskinesia after 5 years from disease onset, anosmia, hallucinations, and dementia [82–84]. A small proportion of MSA patients has an excellent response to L-Dopa and may develop sustained PD-like choreatic peak-dose dyskinesia closely resembling PD, which led to a non-beneficial DBS surgery in one study [85]. The absence of overt autonomic features after 5 years from motor onset strongly argues against a diagnosis of MSA. However, long-duration MSA cases with initial parkinsonian features and very late onset of cardiovascular autonomic failure and bladder problems (after a mean of 11 years) have been reported [86].

Supranuclear vertical downward gaze paresis is highly specific for PSP, but mild reductions in downward (6 to 9%) and upward gaze (20 to 40%) were observed in neuropathologically established MSA cases too [50, 87, 88]. Marked frontal lobe dysfunction with behavioral abnormalities is typical for PSP, whereas in MSA, it usually takes the form of mild executive impairment [83]. Falls in patients with MSA typically occur after a median of 2 years from motor onset, which is later compared to patients with PSP, who manifest with recurrent falls backward starting already in the first year from onset [80, 89]. However, 28% of MSA patients also develop falls within the first year from symptom onset [90]. The leading cause of misdiagnosis MSA in patients with PSP is the presence of cerebellar ataxia. Apraxia, cortical sensory loss, and aphasia are pathognomonic for corticobasal syndrome and have not been described in postmortem-confirmed MSA cases [91].

**Fig. 1:**
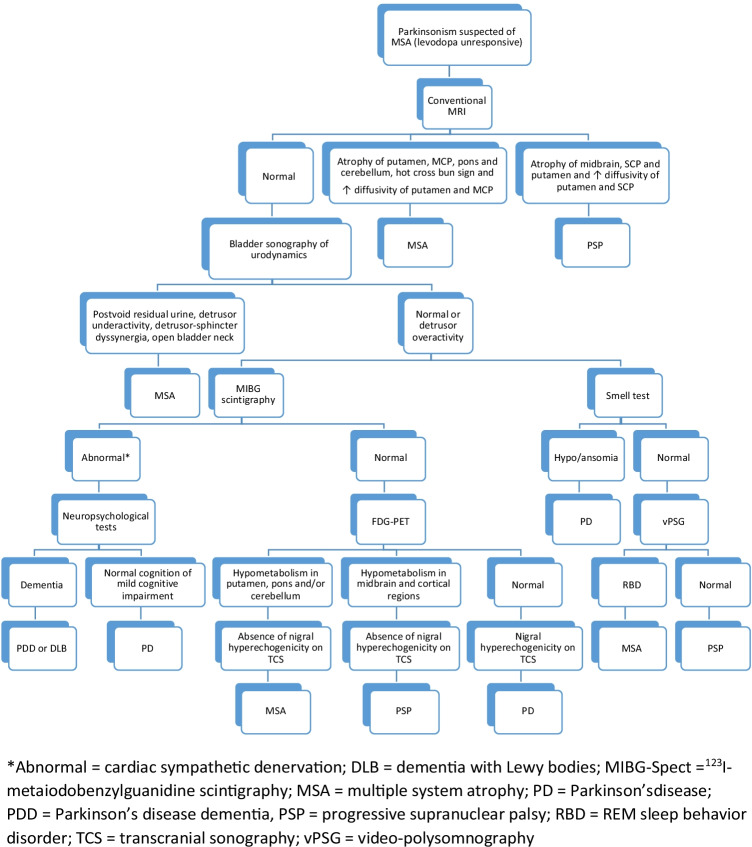
Diagnostic algorithm for a patient suspected of MSA with adult-onset progressive parkinsonism unresponsive to L-Dopa

Time course of cognitive impairment plays a major role in the differential diagnosis of MSA: patients at early stages of MSA never have dementia, while in early DLB, dementia is a core feature. At later stages, both disorders may feature significant cognitive deficits, but these are usually more common and more severe in patients with DLB [92]. Similarly, visual hallucinations do not occur in early MSA and otherwise seldom throughout the disease course (reported overall in 9% of MSA). Fluctuating cognition and profound variations in attention and alertness not related to neurogenic OH are also uncommon in MSA and rather point towards a DLB diagnosis. DLB was the most common misdiagnosis in a clinicopathological study of patients diagnosed antemortem with MSA and the confounding element was in fact the presence of severe autonomic failure in these patients [17]. A provisional algorithm featuring some of the diagnostic tools relevant to the differential diagnosis of a patient with parkinsonism unresponsive to L-Dopa is given in Fig. 1.

### Disorders Presenting with Cerebellar Syndrome

In a patient with suspected MSA-C, SAOA as well as symptomatic, genetic, and immune-mediated ataxia needs to be ruled out.

Symptomatic ataxia due to brain mass lesions and meningeal infiltrates, multiple sclerosis, toxic substances and medications (alcohol, carbamazepine, phenytoin, lithium), HIV, and metabolic disbalance (B1 and B12 deficiency, hypothyroidism) should be considered in patients with a subacute onset of ataxia over weeks or months.

A major differential diagnostic challenge in patients with suspected MSA-C is SAOA, defined as a progressive ataxia with onset after 40 years of age, with negative family history and no established cause of acquired ataxia based on clinical, imaging, and laboratory findings [93]. Patients with SAOA usually manifest additional non-ataxia features such as pyramidal signs and mild urinary dysfunction resembling that of MSA-C. Diagnosis of SAOA is made by exclusion, but a slower progression rate, milder cerebellar features, as well as absence of severe autonomic failure, RBD, and MRI markers suggestive of MSA-C are helpful diagnostic clues [94]. A proportion of patients with MSA-C, who manifest with isolated ataxia at disease beginning and develop autonomic failure only years later may be initially misdiagnosed with SAOA [93]. In the clinical series, the sensitivity of neurogenic OH for distinguishing MSA-C from SAOA at early disease was 32 to 56%; the specificity was 94 to 100%. In more advanced diseases, neurogenic OH sensitivity for MSA-C versus SAOA raised to 64 to 73%, and specificity to 100% [95, 96]. Given that 20% of patients with SAOA may harbor a pathogenic mutation, genetic screening for the most common causes of inherited ataxia in the referral population is required [94].

Positive family history, earlier onset, and slower progression point towards genetic ataxia, in particular autosomal dominant cerebellar ataxia, since patients with autosomal recessive cerebellar ataxia with symptom onset in adulthood usually have a negative family history [97]. Genetic screening should be therefore performed in selected cases with positive family history, early onset (i.e., in the 4^th^ decade of life or earlier), presence of clinical features atypical for MSA (i.e., retinitis pigmentosa suggestive of SCA7 or vestibular neuropathy suggestive of CANVAS), and absence of typical MSA features (i.e., severe and progressive autonomic failure). As mentioned above, genetic testing should be tailored according to the relative frequency of different mutations in the referral population. In European natives, pathogenic mutations should be searched in genes producing SCA1 (*ATXN1*), SCA2 (*ATXN2*), SCA3 (*ATXN3*), SCA6 (*CACNA1A*), SCA7 (*ATXN7*), SCA 12 (*PPP2R2B*), fragile X tremor ataxia syndrome (*FMR1*), SCA17 (*TBP*), CANVAS syndrome (*RFC1*), and Friedreich ataxia (*FXN*) [53﻿]. In Japanese natives, screening for mutations in the *CoQ2* gene should be additionally performed in patients with positive family history [98], as this is the only mutation known to produce MSA. Selected patients with a complex phenotype of movement disorders, dementia, and psychiatric symptoms may be screened for DRPLA (*ATN1*) [53].

Immune-mediated ataxia disorders include paraneoplastic cerebellar degeneration (associated with anti-Yo, anti-Hu, anti-Ri, and other specific antibodies), gluten ataxia associated with anti-transglutaminase-6 antibodies, postinfectious cerebellitis, and primary autoimmune cerebellar ataxia, in which an autoimmune origin is suspected, but there is lack of an obvious trigger or an unclear association with antibodies. This condition may be often misdiagnosed as SAOA [99]. These potentially treatable MSA-C mimics have a subacute onset and rapid progression, and may sometimes be presented with additional clinical features suggestive of underlying malignancy or gluten sensitivity. Other antibody-associated disorders (such as anti-CASPR2, anti-CRMP5, anti-Homer3) may present with cerebellar features, RBD, autonomic dysfunction, and hot cross bun sign [100].

**Fig. 2:**
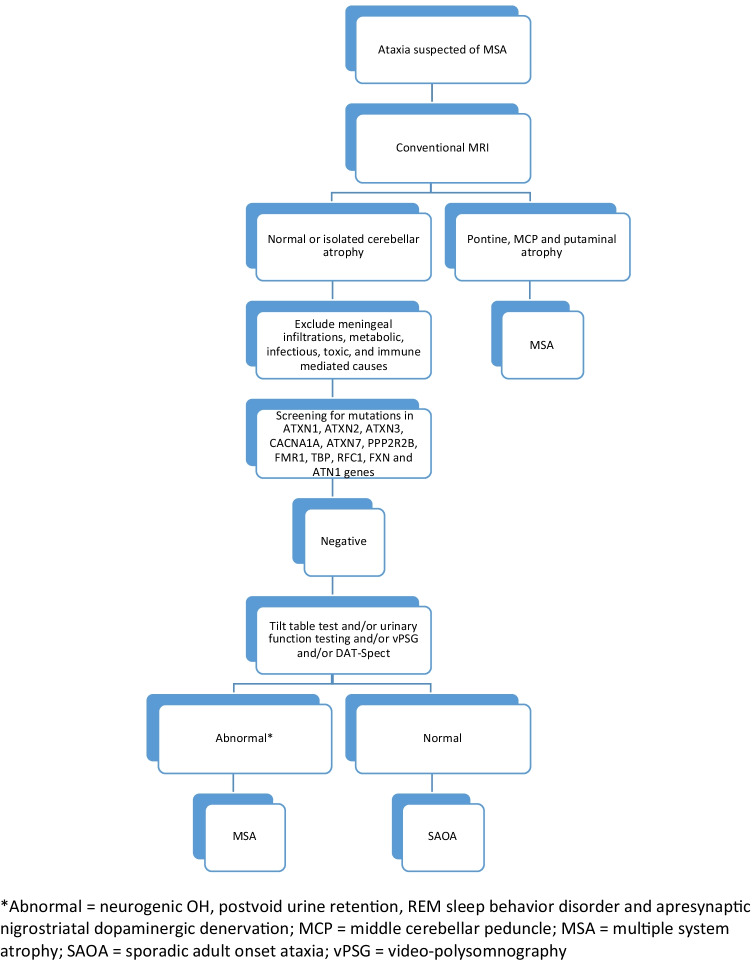
Diagnostic algorithm for distinguishing MSA-C from SAOA

A diagnostic algorithm employing various laboratory tools for distinguishing MSA-C from SAOA is provided in Fig. 2.

### Differential Diagnosis in Prodromal Stages

The diagnostic criteria for prodromal PD [101], DLB [102], and MSA [20] have been recently published and their validation for use in the clinical routine and clinical trials is currently pending. These criteria are designed to identify patients at the earliest disease stage based on the presence of early clinical features.

RBD is a universal prodromal marker of synucleinopathy. A clinically suspect or eventually a polysomnography-proven diagnosis of RBD does not therefore help to differentiate MSA from other α-synucleinopathies, but eventually supports their identification at prodromal disease stages, and the distinction of MSA-P from PSP or corticobasal degeneration and of MSA-C from other causes of cerebellar ataxia [19﻿]. Among prodromal risk factors for PD, polysomnography-proven RBD carries the greatest risk [likelihood ratio (LR) = 130], followed by abnormal dopaminergic PET/SPECT (LR = 43.3), subtle motor signs (LR = 10), and neurogenic OH (LR = 18.5) [103]. Since both polysomnography-proven RBD and autonomic failure are entry criteria for a diagnosis of possible prodromal MSA diagnosis if associated with mild motor signs, the absence of at least one out of anosmia or denervation on cardiac sympathetic imaging, which would otherwise point towards a future phenoconversion to PD, is required [20].

More recently, isolated autonomic failure has been also identified as another prodromal marker of synucleinopathies [21]. Patients with isolated autonomic failure may phenoconvert to MSA, PD, or DLB, and several clinical and laboratory markers have been recently identified as predictors of future phenoconversion to MSA instead of Lewy body disorders including younger age at onset, preserved olfaction, supine heart rate > 70 bpm associated with supine plasma norepinephrine > 100 pg/ml, and orthostatic HR increase > 10 bpm after 3 min [104].

In contrast, criteria for prodromal DLB target the pre-dementia stage and define mild cognitive impairment, delirium, and psychiatric-onset presentations. Although among patients with isolated autonomic failure who later phenoconverted to an α-synucleinopathy with central nervous system involvement, 52% progressed to DLB [104], this presentation was not deemed sufficiently specific to be included in the criteria for prodromal DLB. However, in the context of transition from mild cognitive impairment to dementia, patients who present with parkinsonism and autonomic failure are more likely to be diagnosed with DLB than with other types of dementia [102].

## Conclusion

For a relentlessly progressive disease with no cure such as MSA, an early and correct diagnosis is of paramount importance for a personalized care and timely enrollment into clinical trials with disease-modifying candidate drugs. At the moment, the diagnosis of MSA is still based on clinical grounds despite recent developments in laboratory markers, for which validation in large clinical studies is needed. The new MDS MSA criteria are devised to help an earlier and more accurate diagnosis of MSA, since the certainty of the previous criteria sets was suboptimal. For the first time, the category of possible prodromal MSA is outlined to assist in identifying patients at the earliest stages who might progress to MSA, and who therefore require a close follow-up. The validation exercise on the MDS MSA criteria is currently underway by the MDS MSA Study Group. Future studies should aim for a better characterization of MSA patients by collecting more detailed prospective clinical and laboratory data, including novel imaging techniques (such as the recently announced α-synuclein functional imaging) with postmortem diagnostic confirmation.
